# Conceptualisation of Digital Wellbeing Associated with Generative Artificial Intelligence from the Perspective of University Students

**DOI:** 10.3390/ejihpe15100197

**Published:** 2025-09-27

**Authors:** Michal Černý

**Affiliations:** Department of Information Science and Librarianship, Faculty of Arts, Masaryk University in Brno, 602 00 Brno, Czech Republic; mcerny@phil.muni.cz

**Keywords:** digital wellbeing, artificial intelligence, humanity, autonomy, responsibility, ontological difference, pragmatism, AI, AI literacy

## Abstract

Digital wellbeing has been the subject of extensive research in educational contexts. Yet, there remains a paucity of studies conducted within the paradigm of generative AI, a field with the potential to significantly influence students’ sentiments and dispositions in this domain. This study analyses 474 student recommendations (information science and library science) for digital wellbeing in generative artificial intelligence. The research is based on the context of pragmatism, which rejects the differentiation between thinking and acting and ties both phenomena into one interpretive whole. The research method is thematic analysis; students proposed rules for digital wellbeing in the context of generative AI, which was followed by the established theory. The study has identified four specific areas that need to be the focus of research attention: societal expectations of the positive benefits of using generative AI, particular ways of interacting with generative AI, its risks, and students’ adaptive strategies. Research has shown that risks in this context must be considered part of the elements that make up the environment in which students seek to achieve balance through adaptive strategies. The key adaptive elements included the ability to think critically and creatively, autonomy, care for others, take responsibility, and the reflected ontological difference between humans and machines.

## 1. Introduction

Generative Artificial Intelligence (Abdullah et al., 2022; Benko & Sik Lányi, 2009)—hereafter referred to as AI—represents one of the fundamental phenomena of contemporary education (Chiu, 2023; Condrey, 2023; Fauzi et al., 2023; Rudolph et al., 2023), whose implications for society and the world we live in are not easy to predict, but which are part of numerous reflections (Floridi, 2008; Floridi & Chiriatti, 2020). At this point, it is not our aim to lead a debate on whether artificial intelligence thinks (Floridi, 2023b, 2025), but to analyse the field of relationships and interactions between generative AI and digital wellbeing.

As such, digital wellbeing has been the subject of several research studies (Feerrar, 2020; Nguyen, 2021; Parry et al., 2023; Vanden Abeele, 2021; Waight & Holley, 2020). In general, it can be said that the most common question is how to set up the relationship between humans and digital technologies in such a way that they feel well, that they do not live in the drag of technology (Heidegger, 1967b) or a field with a limited degree of autonomy and ethics (Bridle, 2018), in a space in which they are manipulated by large corporations (van Dijck, 2020; van Dijck & Lin, 2022), but in a space of some freedom, and at the same time they can adequately use technology not to fall into the space of the digital abyss (Norris, 2001; Van Dijk & Hacker, 2003). In this context, it seems clear that it is necessary to understand digital wellbeing also in the context of ethics (Burr & Floridi, 2020a), as it enters the space of human action.

To deal appropriately with AI, humans must possess a certain AI literacy (Cetindamar et al., 2022; Ng et al., 2021, 2022; Wyk, 2024), which some studies have linked to digital wellbeing (Kaya et al., 2025), similarly, it is also possible to see a particular link in competency frameworks such as DigCompEdu (Punie, 2017) and its extension in the field of AI (Bekiaridis & Attwell, n.d.), but there is still a lack of a more robust understanding of how AI enters the phenomenon of digital wellbeing.

In this study, we treat digital wellbeing as a dynamic socio-psychological process (Vanden Abeele, 2021). Digital wellbeing depends on many factors, such as self-realisation and learning activity (Shao et al., 2024), but it also depends on specific social conditions and contexts (Nguyen & Hargittai, 2024). The objective of digital wellbeing is to promote the responsible use of digital technologies that enhance, rather than diminish, the quality of life for individuals and society, enabling people to lead fulfilling lives alongside technology (Burr et al., 2020). This involves the ability to analyse risks and integrate them into the process of achieving personal wellbeing, while taking into account issues of sustainability (Moşteanu, 2020), ethics (Burr & Floridi, 2020a) and AI literacy (Filep et al., 2024; Kaya et al., 2025), as well as other environmental factors. It is not a permanent phenomenon, but one undergoing constant change concerning changes in the individual’s technology and societal contexts, development and needs. In this study, we will understand digital wellbeing as a dynamic balance in a person’s interaction with digital technologies, which is subjectively perceived as good in all dimensions of human existence. We thus integrate the approaches of Vanden Abeele (2021) and Burr and Floridi (2020a).

### The Relationship Between Digital Wellbeing and Artificial Intelligence

Kaya et al. (2025) highlight the association between AI literacy and digital wellbeing, noting that individuals with higher levels of AI literacy also have higher levels of digital wellbeing. In the specific context of the hospitality industry (Buhalis & Leung, 2018; Filep et al., 2024), talk about the positive impact of AI on guests’ digital wellbeing, keeping in mind that this is an extension and improvement of service, not a specific effect on a broader sense. Other positive aspects of the relationship between AI and digital wellbeing can be found in the field of mental health care, where we can see applications from Ellouze and Hadrich Belguith (2025), who highlight chatbots that offer individualised mental health support, or Y. Kim et al. (2025), who focus on cognitive training using generative artificial intelligence for adult users.

Bond et al. (2025) ask a fundamental question in their study, namely what impact AI will have on digital wellbeing, referring to the foundations of positive psychology (Csikszentmihalyi, 1997; Seligman, 2011) and highlighting the fact that AI may lead to limitations in the ability to achieve goals with reasonable effort (flow, PERMA model (Seligman, 2011)) or more generally to forms of meaningful goal fields. They draw attention to the fact that these systems can directly impact wellbeing, precisely because of the loss of meaningful, fulfilling activities and tasks. This also corresponds to some extent with studies related to the phenomenon of labour market transformation (Eloundou et al., 2023; Webb, 2019; Zarifhonarvar, 2023), where we can talk about fundamental transformations (Frey & Osborne, 2017), which deprive some people of an essential constitutive element of their security and identity (Bauman, 2013).

There are a large number of negative views on the impact of artificial intelligence on various areas of human society in social discourse, such as environmental factors (Zewe, 2025), fake news and deepfake (Esezoobo & Braimoh, 2023; Sharma & Kaur, 2022), hallucinations (Alkaissi & McFarlane, 2023; Elias & Alija, 2023), and plagiarism (King & ChatGPT, 2023), which also does not contribute to building a sense of wellbeing (Roffarello & De Russis, 2019; Seligman, 2011). Many authors now refer to issues of ethics and academic integrity (Al-kfairy et al., 2024; Zlotnikova et al., 2025), which play a significant role in the phenomenon of digital wellbeing in the context of generative AI.

Technological transformation is making the world we live in increasingly complex and abstract (Flynn, 2007; Webster, 2014), fluid (Bauman, 2013) and threatened (Beck, 2009) of crisis (Haider & Sundin, 2022; Latour, 2018, 2021). The impacts of technology on the wellbeing of young students have been described as fundamentally negative, at least as far as the comprehensive UNESCO report by West (2023). In this field of thought, generative AI outside of specific therapeutic applications does not seem to have the possibility of positively impacting student wellbeing. Yet it is a technology that is there (Abdullah et al., 2022; Balmer, 2023) and cannot be turned off or avoided. As B.-J. Kim and Lee (2024) point out, how AI is implemented in the institution’s functioning in relation to the role of self-efficacy is quite crucial.

The recently established domain of education and psychological interactions associated with generative AI exhibits an ambivalent character within the context of literature (van der Maden et al., 2024; Trocin et al., 2023). However, it appears uncontestable that, in terms of specific approaches, measures or policies, this subject will necessitate particular forms of research and application reflection, as evidenced by the case of social media (Virós-Martín et al., 2024), given the significance and distinctiveness of its forms of interaction. Consequently, we advocate for a dedicated exploration of the nexus between AI and digital wellbeing, emphasising its relevance in our research agenda. Specifically, the literature pays particular attention to the ethics of designing AI systems for dialogue with humans (Balcombe & De Leo, 2022; Floridi, 2023b); problems with dynamic interaction (Dutta & Mishra, 2023), which in many ways resemble human dialogue; or emphasising that digital wellbeing is contextual and that generative AI shapes specific contextual frameworks in which consideration of this topic must be reflected upon (Gennari et al., 2023; Nguyen & Hargittai, 2024).

At the same time, it can be said that a more systematic reflection on the relationship between generative AI and the digital wellbeing of university students in a more general perspective is lacking, i.e., how the existence of different tools and the pressure to work with them impacts their digital wellbeing. This research study attempts to address this knowledge gap and contribute to its closure. The research focuses specifically on LIS (Library and Information Science) students at the university; consequently, it has limited possibilities for generalisation. However, it has a qualitative character that enters the discussion regarding conceptualising the entire phenomenon at the intersection of pedagogy, psychology, information science, and philosophy. The objective of the present study is not to fully address this gap, but rather to provide a continuation of the discourse on this matter (Burr & Floridi, 2020a; Vanden Abeele, 2021), with a particular focus on the needs of the students themselves.

## 2. Methodology

The study’s introduction states that the relationship between generative AI and digital wellbeing is relatively new. Therefore, this study attempts to shape a possible framework or conceptualisation of the topic at the higher (university) education level.

For this reason, the study works with a qualitative research design, specifically a thematic analysis (Braun et al., 2019; Braun & Clarke, 2006; Clarke & Braun, 2014). The research aims not to describe the contribution of individual phenomena or their correlations to digital wellbeing, but to create a framework that can be further used and developed educationally. We take a qualitative research approach because of the ambition of creating a new theory.

Research question: How do LIS students reflect on the relationship between their digital wellbeing and generative AI? To elaborate further, what are the values, approaches, and topics incorporated into the rules recommended for generative AI in the context of digital wellbeing? At the same time, we attempt to complement and extend this question through the lens of educational psychology: what phenomena enter the field of conceptualising digital wellbeing and generative AI, and how can they be responded to educationally? This study does not aim to create a concept for regulating artificial intelligence or the school environment. Instead, it seeks to provide a broader perspective on how this phenomenon can be considered in the context of higher education.

As described below, we use a semi-indirect method of analysis and quantitative research, corresponding to common research practice in the context of the phenomenon under study (Cecchinato et al., 2019; Peters & Ahmadpour, 2021).

### 2.1. Research Sample

The research was conducted in the context of the Digital Competence course taught in the spring semester 2025 at the Faculty of Arts. It is a required course for students in the field of Library and Information Science (LIS), which is designed for the second semester of study. 71 people were enrolled, of whom 64 submitted the assignment and thus belong to the research sample. The university does not record the gender of the students. Of the 71 persons, 5 were in combined studies and 66 in full-time studies: 12 studied LIS as a double major and 59 as a single major. All participants were adults—the typical age of students in full-time study is between 20 and 22. Students in combined study programs are mostly employed and older. University rules do not allow for tracking the numbers of women and men, but it can be said that women make up the majority of the sample. Participants did not receive any compensation for their participation in the research.

The LIS education curriculum often includes digital competencies and the relationship to the information environment (Bawden & Robinson, 2022; Bright & Colón-Aguirre, 2022; Kizhakkethil & Perryman, 2024). Students are confronted with artificial intelligence from the first semester onwards (in this research, they usually work with it in their second or third year), and a higher level of digital competence and experience influences their view of the issue. On the other hand, this position is interesting for our research because it allows for a deeper and more structured insight into the issue.

At the same time, we are aware that the data obtained may reduce the generalizability of the results, but this is not the goal of qualitative research. The research aims to find answers to research questions that are integrally linked to our chosen population (LIS students, whose level of information literacy, as well as knowledge related to digital skills and artificial intelligence, is higher than that of the rest of the population).

### 2.2. Research Instrument

The research tool aimed to capture essential values or moments associated with working with generative AI. In this study, we specifically focus on the broader concept of digital wellbeing. Thematic analysis of written student assignments to evaluate students’ attitudes towards various aspects is a customary element of the assessment process. Rude and Frenzel (2022) indirectly analyse attitudes towards collaborative learning, while Nabor et al. (2024) analyse attitudes related to online learning and writing. The most closely aligned with our research is the study by Mueller (2017), which captures students’ attitudes associated with reflecting on their own experiences of teambuilding courses at university through thematic analysis. Consequently, the present study contributes to this established body of research.

Students were given the following assignment: *Try to formulate ten principles or rules that users should follow regarding safety, conservation, efficiency and digital wellbeing when working with generative dialogical AI. For this assignment, please list the five sources you have consulted.* The assignment was more loosely worded verbally, the items were seen as more indicative, and the number ten was also not strict. Some assignments had 8, others 12 points or 10 points, some just listed rules, and others combined rules and textual descriptions. This variety is visible in the results presented. The students worked on the assignment at home, so it is possible that they used generative AI to create it. This limitation is an integral part of the research procedure.

Some students met the required references to sources, but not others. They aimed to try to ground particular points in particular sources or contexts that offered at least a basic technical depth of reasoning or the possibility of defining themselves against commonly existing approaches.

The linking of rules relating to behaviour and thinking is a key aspect of the theoretical analysis in the study. We draw on concepts from pragmatist philosophy, which emphasizes that thinking and acting form a single interconnected whole (Dewey & Bentley, 1960; Šíp, 2019)—thus, from an analysis of the conceptualization of action (the ten), mental structures in the minds of students can be inferred without having to differentiate between rational and emotional fields or conscious and unconscious (Damasio, 1994; Dewey, 1934; Johnson, 2017; Lakoff & Johnson, 1999). Thinking and acting form a unified style in pragmatism that can be consistently examined.

This broader assignment facilitates a more comprehensive survey of the conceptualisation of digital wellbeing among students. Concomitantly, it is recognised that the data would enable a more expansive conceptualisation of select phenomena or examine other subjects with greater rigour. It is acknowledged that the conceptualisation of digital wellbeing is being constructed indirectly, yet this is being done according to the principles associated with a pragmatic philosophical framework that links thought and action.

Since our research works with a one-time collection of text responses, we do not have the tools for triangulating the research. At the same time, however, we can say that the sample size is considerable for qualitative research (64 people, which corresponds to 90% of enrolled students), and the number of research units (474) is sufficient for quantitative research too. However, this research is strictly qualitative in nature. The sample was saturated.

### 2.3. Data Collection and Processing

The assignments were submitted to the university information system in DOCX or PDF formats from 6 March 2025, to 14 April 2025. This time is crucial to reflect on the social and technological context in which the responses were generated. The tasks included working with literature on rule formation that was removed from the research sample. Of the 64 files submitted, 61 were used and met the assignment. These were not interviews, but written responses—“ten commandments”—unrelated to personal data.

In total, we worked with 61 student responses in the document, which were coded with 474 codes and further categorised as shown in Table 1, corresponding to about 7.7 codes per student assignment. It is necessary to say that some statements or rules fall into more than one category. Some categories are further differentiated into subgroups, especially related to our text’s actual aim, i.e., the transformation of digital wellbeing in a broader sense.

The choice of categories corresponded to an open coding based on repeated document readings. This process produced the categories shown in Table 1. Some relevant to this study’s research purpose were further differentiated into sub-categories. The **Thinking** was divided into the following units: AI as an enabler (13), creativity (6), critical thinking (30), limits of AI (19), responsibility (17), miscellaneous (6). **AI literacy** included the following categories: Effectiveness and prompting (20), AI principles (17), education (17), and miscellaneous (8). **Ethics** included the following categories: hateful and false content (24), plagiarism (8), transparency (24), and miscellaneous (23). **Wellbeing** included the following subcategories: time and boundaries (39), inhuman actors (17), other specific measures—outside of time (8), and miscellaneous (12). These subcategories are partially reflected in the section presenting the results, which is therefore not a random illustration, but is based on the subcategorical classification of statements in the research sample.

On the other hand, some expected categories from the literature—such as reflection on distance, relationship to design, and ethical design of language models—were not represented in the sample at all. The categories listed in Table 1 are structured into three areas presented in the results, but at the same time, the individual subtopics correspond to the content under the individual codes—digital wellbeing in the narrow sense; the relationship between AI and thinking in the context of wellbeing; and environmental aspects.

Within the broader framework of the entire set of statements, the categories that are most densely represented are thinking (19%), ethics (16%), wellbeing (15%), and AI literacy (13%), which corresponds to the literary discourse concerning the specified subjects, while concurrently being related to the very construction of the categories. The least represented category in our research is security—general (2%). The reason for this is probably the specific transfer of the topic of security to data and information protection, or the need to verify information. We left this category separate for two reasons: (1) In the Czech environment, the topic of security at the national policy level is strongly linked to digital wellbeing and therefore has local significance. (2) It forms a relatively cohesive whole closely linked to the national narrative. At the same time, it is necessary to admit that some definitions of digital wellbeing do not work with security and perceive it as a separate topic. Given the qualitative nature of the research, the density of codes is not a determining factor in the relevance of the research findings.

The miscellaneous was used to shape the structure of the results. The ‘Uncategorised’ section includes statements that cannot be categorised under existing subcategories. These statements are still relevant to the theme of the category to which they belong. As this is a thematic analysis, the numbers are provided merely to illustrate the coverage of individual statements. Due to the sample size and the research’s qualitative nature, we are cautious about conceptualising density as a quantitative parameter. The study aims to create a qualitative model of the subcomponents of digital wellbeing related to generative artificial intelligence, rather than quantifying individual phenomena.

Thus, the research relied on conventional content analysis using open coding. The coding process involved several stages of repeated reading. One researcher read through the statements repeatedly, gradually creating codes inductively. After some time, the researcher coded the statements again, resolved any code inconsistencies, and refined their descriptions. Although the coding process is subjective due to the epistemic lens of a single researcher, the consistency of the coding was maintained. The individual statements are treated as atomic; therefore, we do not indicate the numbering of respondents. Atlas.ti 24 software was used for coding and data processing.

One statement can be marked with multiple codes, as shown in Table 2. The table demonstrates a strong connection between statements linking thinking and AI literacy (6) and thinking and wellbeing (8). In general, it can be said that the Thinking category has the most statements in common with other categories due to the nature of the phenomenon under investigation.

Thematic analysis provides the foundation for the subsequent step of the analysis, which involves the development of a model grounded in the principles of grounded theory (Goldkuhl & Cronholm, 2010; Walker & Myrick, 2006). The data obtained was then subjected to a process of breakdown, coding and division into categories. This was followed by a process of abstract lifting, during which attempts were made to identify connections between the individual perspectives, or rather, a standard model that would explain the formulation of particular rules. Consequently, strong theoretical assumptions (a priori) are not employed in our analysis or research tool, even though our work is conducted within a field influenced by theories about digital wellbeing, both as researchers and as respondents to the study (Dunne, 2011).

## 3. Results

### 3.1. Digital Wellbeing in the Narrow Sense

In terms of **digital wellbeing** itself, the responses can be differentiated into three main areas. The first—the simplest—is the phenomenon of boundary setting. Students accentuate the topics of time spent on devices or with AI-enabled tools and services as a key theme in this area. Significantly, they do not give specific time figures, but talk about general **limits and boundary setting**:

*Don’t spend all your time on AI.*

*Set a time limit on using the tools and be mindful of your mental and physical wellbeing.*

*Don’t let working with AI consume you entirely. Set limits on your time spent in the virtual environment.*

*Think about screentime and its distribution during the day*

Students accentuate the difference between digital and physical environments, and we can see recommendations based on this distinction:

*Excessive interaction with AI tools can lead to digital stress or fatigue. Set limits and engage in activities outside the digital world.*

*AI will talk to you for hours and is available around the clock. But don’t overdo it; learn to turn off the tools in time. Sufficient offline time is essential for your mental wellbeing.*

There are also comments related to the topic of addiction, perhaps more specifically, the addictive behaviours that can arise through interaction with AI:

*Addiction to AI can easily arise. So, we should not rely too much on language models for personal relationships or emotions.*

*Create healthy habits: Approach AI as an everyday tool, but don’t let it become an addiction. Scheduling regular breaks from technology will help maintain balance.*

*Beware of addictions. Talking to AI can take a toll on your psyche.*

These times can easily be summed up by students identifying AI as a tool that can negatively influence their digital wellbeing—from an extreme version of addiction to the plane of some absorption or discomfort. It also seems that one of the essential principles is precisely setting limits on the use of AI and rationalising the whole interaction between humans and technology:

*Set time limits for working or playing with AI—as with social networking, digital overload can occur.*

*Promote digital wellbeing—Balance time with AI technology with offline activities. Prolonged use of AI can lead to digital fatigue or impaired concentration.*

Even from a simple frequency analysis, but especially from the content of the individual statements, it is clear that digital wellbeing is an essential topic for students. They are looking for a way to develop a healthy relationship with AI that is not entirely dismissive (because it generates other problems), but also not uncritically positive. A second important theme in this category is reflecting on a certain anthropomorphisation of AI, i.e., the realisation that **AI is not human**, which can be difficult at the level of psychological and social perception:

*Don’t use AI as a substitute for personal interaction—Using AI is excellent for a variety of tasks, but don’t forget the importance of personal contact and interpersonal relationships.*

*Be critical of how AI interacts with you. AI may seem human, but it’s not a real person; don’t form emotional attachments to it.*

*Don’t confuse a conversation with a generative AI with interacting with a real person. It may seem empathetic, but it doesn’t understand emotion or context. It can mislead us with unrealistic interpersonal relationships.*

*AI may be pleasant, non-judgmental and always available, but it is not human. If AI replaces real contacts, you may gradually lose social skills, empathy or confidence in communication. Maintain natural contact with the people around you.*

These statements reveal three critical phenomena. The design of applications using text-based generative AI is such that they try to create the appearance of human communication. Students point out that such a practice is not okay, but pleasant and appealing to humans. Thus, we are faced with an interesting situation of enchantment or an offer of interaction that everyone knows is deceptive but seductive at the same time. The second moment is forgetting—communication can easily be framed initially as standing-man, but through design, forgetting can easily occur in a particular interaction about this fact. The third phenomenon relates to the manifest imperative of the importance of human and social contact, as also illustrated by the following statements:

*AI should not play the role of our friend, therapist or otherwise close person. Social contact is essential to us and should not be replaced by chat shoes.*

*Keep the human dimension—Remember that AI is a tool, not a replacement for human creativity, intuition and decision-making. Use AI as a helper, not as a substitute for your judgment.*

Statements related to humanity and empathy are key to wellbeing:

*Don’t expect human understanding—AI can simulate conversation but doesn’t experience emotion or perceive context like a human. Use it as a tool, not as a substitute for interpersonal communication.*

*Although it may seem empathetic or “human,” AI has no emotions or intentions. Let’s keep a healthy distance.*

*AI cannot replace humans in terms of empathy and the emotional side.*

*AI can be persuasive, sometimes even human. It can respond with humour, compassion and empathy, but it is all just the result of computation. It cannot understand, feel or take responsibility. Knowing that you are communicating with an algorithm helps you avoid unrealistic expectations and emotional attachment.*

These references can be read on two levels—the first is the inherent manifestation of the uniqueness of human relationships and their social meaning, which cannot be replaced by technology, which cannot have compassion or emotion because it has limited time and resources, attributes that make human relationships valuable. The second level is the question of why learners mention these aspects at all—here it seems to be (to some extent) a projective issue that needs to be acknowledged. The relationship with technology is easier and safer; it does not require special care and does not carry the risk of ‘failure’. Thus, students need to realise in some respect that contact with a human being is essential for wellbeing, and this is true at the whole level of the relationship, both in the social interaction provided and received.

In some ways, a different narrative is presented by the recommendations that seek to emphasise a certain **autonomy and freedom of the person in the technologies** they use, and this freedom can be secured by emphasising reflective skills, self-perception:

*Define when and how you use AI. Automate routine tasks but retain human decision-making where empathy and moral judgment are needed.*

*Reflect on your relationship with AI. Everyone has their take on it, everyone uses AI for different things, at other times, with varying communication styles. The main thing is to realise what applies to you! Find your style, or feel free not to use AI. The main thing is your comfort.*

*Always know why you use AI and don’t waste time on aimless questioning.*

Another essential facet may be the fear of **humans being replaced by technology** (we’ll come back to this topic). Still, as in the previous examples, we can see an effort to emphasise the irreplaceability of humans while emphasising their feelings and mental health, which may be compromised by technology:

*AI can complement your work, but it should not completely replace it.*

*AI should help, not replace human thinking. Important decisions should always be thought through and thoroughly verified.*

*Be aware of how AI affects your emotional health, concentration and relationships. Too much reliance on AI can affect your wellbeing and self-confidence. Keep in mind the impact of AI on society and interpersonal relationships.*

### 3.2. The Relationship Between AI and Thinking in the Context of Wellbeing

Our study aims not to explore all aspects of the transformation of the conceptualisation of thinking, but rather to highlight some fragments that we see as crucial in conceptualising digital wellbeing. The first area we would like to build on the last two parts of the previous analysis concerns the role of AI in human relationships. In our research, the level of AI as a helper, as a specific component that helps but is explicitly subordinated to the human, has proved to be strong, whereby the student (or the human in general) retains its **ontological value** and position in the “order of creation”, if we can borrow this theological term:

*AI can be a great helper, but users must still actively develop their thinking and creativity. AI should complement human creativity, not replace it. Human creativity, empathy and originality are irreplaceable.*

*The user should only use AI when necessary. Tools should only play the role of an assistant.*

*Use AI to improve efficiency—Use technology only for tasks that help you, and don’t think you have to spend all your time with AI.*

*Set a clear goal, formulate questions thoughtfully, and organise your deliverables—you’ll save time and energy. The user should think of generative AI as a tool supporting thinking and creativity, not as an authority or a substitute for their judgment.*

These points accentuate two important realities related to digital wellbeing—firstly, the aforementioned ontological superiority, as AI should do what humans want it to do. As much as it doesn’t always know exactly what it wants, a distinct sense of superiority is manifested here, creating a simultaneous sense of freedom. The second important point, which is educationally exciting, is the question of the **delegation of activities**. Students have a relatively strong awareness that through their activity they learn, are educated, successful and valuable, that through activity they become better and develop, and at the same time, that AI can help them with many things. The primary task of education seems to be to find ways to deal with this tension—on the one hand, the possibility of automation and delegation, on the other, the emphasis on work and activity as a source of ontological creation of humanity.

Another important point related to digital wellbeing is ambivalence about what AI can and cannot do. There are manifested capabilities of generative tools, often associated with an emphasis on transforming the working species. Still, at the same time, the technology’s fundamental limits must be kept in mind when interacting or collaborating with it. This creates an ambivalent field in which, on the one hand, there is the present risk of replacing humans with cheaper and more powerful technology or the feeling of communicating with an intelligent partner. On the other hand, there is a growing set of demands on the human in this asymmetrical communication, which must be written into the perceived digital wellbeing.

An example is the pressure for **responsibility, which always belongs to the human being**:

*AI does not have consciousness, opinions or human values. It is essential to distinguish between a cue and a decision—that remains up to you.*

*Holding humans accountable for the content they create.*

*AI can be a great help but doesn’t replace your creativity, judgment, or ethical responsibility.*

*We shouldn’t use AI for critical issues, but our minds. We should properly combine our experience and knowledge with AI’s answers.*

*You are still responsible for your decisions, not the AI.*

Artificial intelligence is thus perceived as productive but irresponsible. The first answer may seem optimistic, i.e., AI can be a kind of advisor or, as mentioned above, a helper. However, at the same time, there are a significant number of statements (from almost all learners) who simultaneously say that what AI produces is **not** automatically acceptable and **accurate**, that it must be constantly subjected to scrutiny and evaluation:

*AI may not always be right. Facts need to be verified from multiple sources.*

*Generative AI can produce false or fabricated information. Always verify information.*

*Verify that not all sources on which AI bases its answers are relevant, and even when it does cite sources, its accuracy cannot be entirely relied upon.*

*Check the facts: AI can be persuasive, but not always accurate. Verify necessary information from trusted sources.*

These statements are undeniably factual, but when viewed through the lens of digital wellbeing, they are deeply troubling because realistically, it is not possible to check and recheck all answers constantly and to be held accountable in a situation where the ‘other’ is not the child or pupil in whom one invests this form of care and responsibility with an eye to the public good, but also a threat or competitor. The students’ statements imply they are forced to empower the *dragon about to devour them*. Such a state of affairs cannot lead to long-term equilibrium; however attractive or appealing it may be, it may be in many ways in the short term.

At the same time, we can talk about the risks associated with AI, which students talk about as being associated with an inevitable erosion of the quality of human thinking that is carried by the **creativity** that we should retain:

*Don’t rely on AI with everything: Learn beyond it—develop your skills, knowledge and creativity without assistance.*

*AI can inspire, but we can’t let it diminish our creativity and creative process.*

*We shouldn’t use AI as a substitute for our creativity, but only as an auxiliary supplement to complement our minds.*

These points accentuate another critical aspect of working with AI that we have already written about—using AI to produce a specific type of output threatens the human mindset, the education of learning, as one student puts it:

*AI can support creativity and analysis, but should not replace one’s judgment, study or research.*

The education system should provide an environment where education and work with AI are balanced adequately and reflectively. Students manifest concerns about the impact of AI on their thinking, creativity, and personality while living under pressure to work with AI systems systematically, to compare themselves to them, or to look for specific elements in which they are unique. Such a system compromising the integrity of one’s academic scholarship cannot lead to digital wellbeing in academia. It is therefore not surprising that the demand for **transparency** in the use of AI, or warnings against plagiarism (8), is by far the most frequently heard as a key element of ethical work with AI (24 in total)—as with time limits where appropriate, we see an attempt to find some coping mechanism for AI and to banish it to certain limits or boundaries:

*Be transparent: If you use AI to create content, inform others about it and do not try to pass off AI output as your work.*

*If the output you’re sharing was created (in whole or in part) with the help of AI, it’s a good idea to make that clear. This ensures fairness and credibility and helps spread digital literacy in the community.*

*We should be transparent if we use the content generated. We are ensuring fairness to others.*

*Acknowledge if you’ve used AI to write text or help you with an assignment.*

In some ways, transparency can also be seen as a form of demand for differentiation—separating the human and non-human, the human and the technical, and naming them clearly, may be one of the keys to how we approach digital wellbeing. If such a form of separation could be found, it would arguably also create a much easier interaction framework that would anthropomorphise generative AI less.

### 3.3. Environmental Aspects

In our research, it became clear that not only the topics of individual access to technology, i.e., the individual perspective, are essential for students, but also that aspects related to social and environmental issues play a significant role. However, as they do not fit into the core of reflected digital wellbeing, they can play an essential role in the overall perception of generative AI and its use, as we will show in three areas that emerged from our research.

The first area is the relationship between generative AI and **hate content**, i.e., the social environment. Students believe that technology should play a role that is integrative and cohesive in society rather than contributing to its polarisation. It is the polarisation of society, the creation of hateful content coupled with the breakdown of social ties, that ultimately has a potentially significant impact on digital wellbeing, for example, through social media:

*Don’t use AI to create violent, harmful or explicit content.*

*Don’t abuse AI: Don’t use AI to develop harmful, hateful or manipulative content.*

*AI tools should not be used to create hate speech or offensive or discriminatory content.*

These examples point to a particular risk of social-emotional manipulation. When texts about a divided society appear in the literature, students also project their concerns about unregulated artificial intelligence in this direction. At the same time, this theme quite naturally links to the theme of the breakdown of the accurate picture of the world—we have already referred above to aspects of the need to verify information generated by AI, where we have suggested that this is more about technical error or imperfection on the part of the technology. However, the students point out that AI can be used to deliberately **create the dissemination of false information** and thus transform the information environment:

*Don’t use AI to generate illegal content or fake news.*

*Do not use AI to deceive, mislead or spread misinformation.*

*Do not use AI to create hoaxes, fraudulent news or manipulative text or images.*

*We should never use AI to spread misinformation, deception or manipulation. We must respect copyright and ethical principles.*

The creation of these false information structures again leads to an inevitable weakening of society, its manipulation or unfreedom. It seems necessary to emphasise the need, which appears to be restated, to seek educational and social mechanisms for developing the resilience of society, which would make it possible to be immune to these threats. However, the students named another aspect of the functioning of generative artificial intelligence that relates to the environment in terms of **ecology and sustainability**:

*Avoiding the overuse of AI—especially in large-scale and repetitive tasks—due to the high power consumption of data centres.*

*Generative models consume significant amounts of energy and water, so they should only be used when necessary or efficient.*

*AI should be our last resort for querying and information retrieval. Using AI puts a burden on the environment, and its use is often not even necessary.*

*We should use AI judiciously and only when its contribution is needed to protect our planet.*

We can see three essential components in these rules or statements—it’s inherent emphasis on the environment and its protection. At the same time, there is a dimension of a certain artificiality (σωφροσύνη), i.e., a call to look for where AI is needed and when it is not. It is a component that shows that a particular paradigm of playing with AI or non-binding testing is ethically problematic. At the same time, there is also an emphasis on social ethics, the dimension of the need to reflect on one’s actions not in terms of the individual alone, but to understand them as part of a broader framework.

### 3.4. Dynamic Model of the Relationship Between Digital Wellbeing and AI

By analysing students’ responses, we can create two models showing the relationships between responses as a conceptualisation of digital wellbeing in the context of generative AI. The construction of these models was informed by established theoretical frameworks. The individual areas were rearranged and analysed as they were coded (see Table 1 and Table 2). The aim was to search for a certain model of continuity and common elements that would allow for the creation of a more coherent model (Walker & Myrick, 2006) in the context (but not in the a priori assumption) of the current state of discussion and knowledge about digital wellbeing (Dunne, 2011).

**Figure 1:**
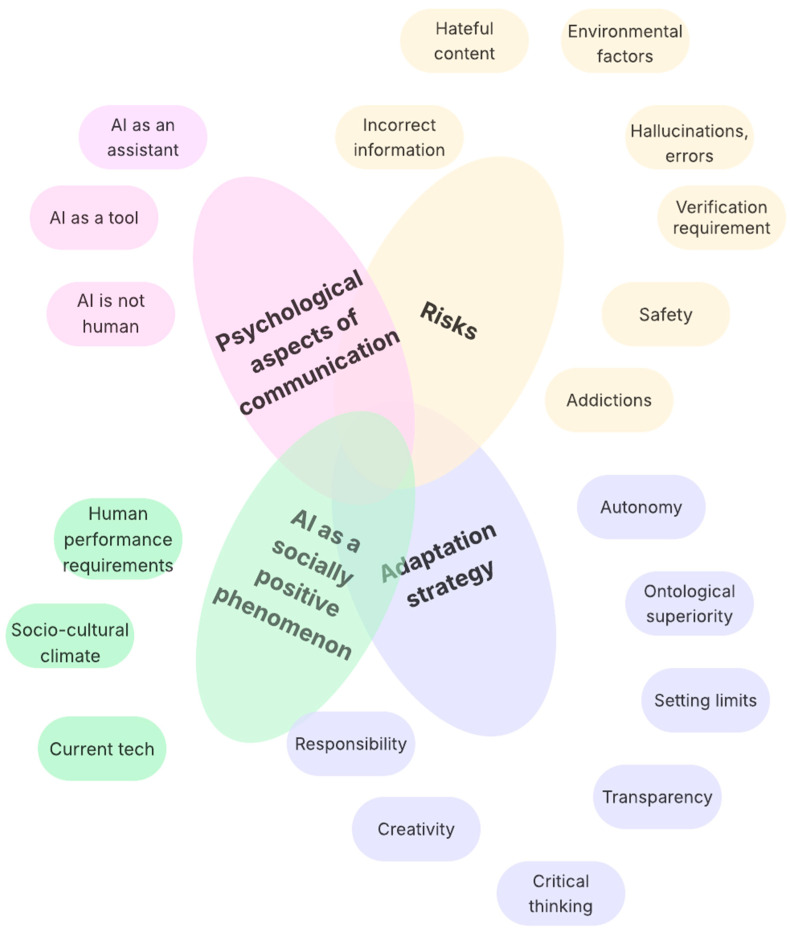
Four-dimensional model of digital wellbeing of human and AI interactions based on examples provided by students of LIS. While the four dimensions are relatively robust and stable, the examples may depend heavily on specific students.

Figure 1 captures the understanding of digital wellbeing as a four-dimensional phenomenon, in which factors related to risk reflection, psychological aspects of communication with inanimate technical systems with AI, adaptation strategies, and the perception of AI as a socially positive and desirable phenomenon come into play in the context of working with artificial intelligence.

Analysing the results reveals specific links between the individual categories, suggesting the potential for a model of digital wellbeing in the context of human interaction and generative AI.

The first area is the psychological aspects of communication, which reveal differences in users’ approaches to AI. Some perceive AI as a non-human communication agent, some as a highly non-autonomous tool over which humans have complete control, and some as an assistant. These three concepts are reflected in different interaction methods, levels of trustworthiness and feelings associated with generative AI.

The second group of responses relates to how these communication propositions can be applied to the coexistence of humans and AI. Themes include the need to preserve human authenticity and a certain ontological primacy of humans (i.e., emphasising that humans are more important than AI and that AI should serve their needs, not the other way around), setting limits on how to work with AI, and preserving the quality of humanity, which is associated with responsibility, critical thinking and creativity. In working with generative AI, digital wellbeing must save humanity and separate humans from technology. In light of the statements in the first category, it can be concluded that AI is not a friend, but rather a dangerous tool or competitor.

The third group of phenomena contributing to digital wellbeing relates to risks students know well. These range from providing incorrect information and hallucinations to the need to verify information, issues of safety, hateful content and addictive behaviour. These factors alone would lead to a rejection of working with AI or great scepticism about its use.

In the context of risks, it might seem that students reject AI because it threatens their humanity, freedom, and wellbeing as such. At the same time, however, they show that wellbeing does not consist in “doing nothing,” but is indisputably linked to the need to lead a good life, which requires activity. This is reflected in the adaptation strategies discussed (creativity, critical thinking, responsibility, autonomy) and the motivations for using AI despite the risks.

In the context of risk, it may appear that students reject AI because they feel it threatens their humanity, freedom and wellbeing. At the same time, however, they demonstrate that wellbeing does not consist of ‘doing nothing’ but is indisputably linked to the need to lead a good life, which requires activity. Despite the risks, this is reflected in the adaptation strategies discussed, such as creativity, critical thinking, responsibility and autonomy, and the motivations for using AI. Students cite the socio-cultural climate forcing them to work with AI, the importance of adaptive strategies for digital wellbeing, and social pressure associated with research and current technologies. The limitations and imperfections of these technologies are ambivalent, creating an opportunity to preserve a particular form of humanity as a source of performance and power, but also leading to unpleasant feelings associated with using AI tools that generate errors.

**Figure 2:**
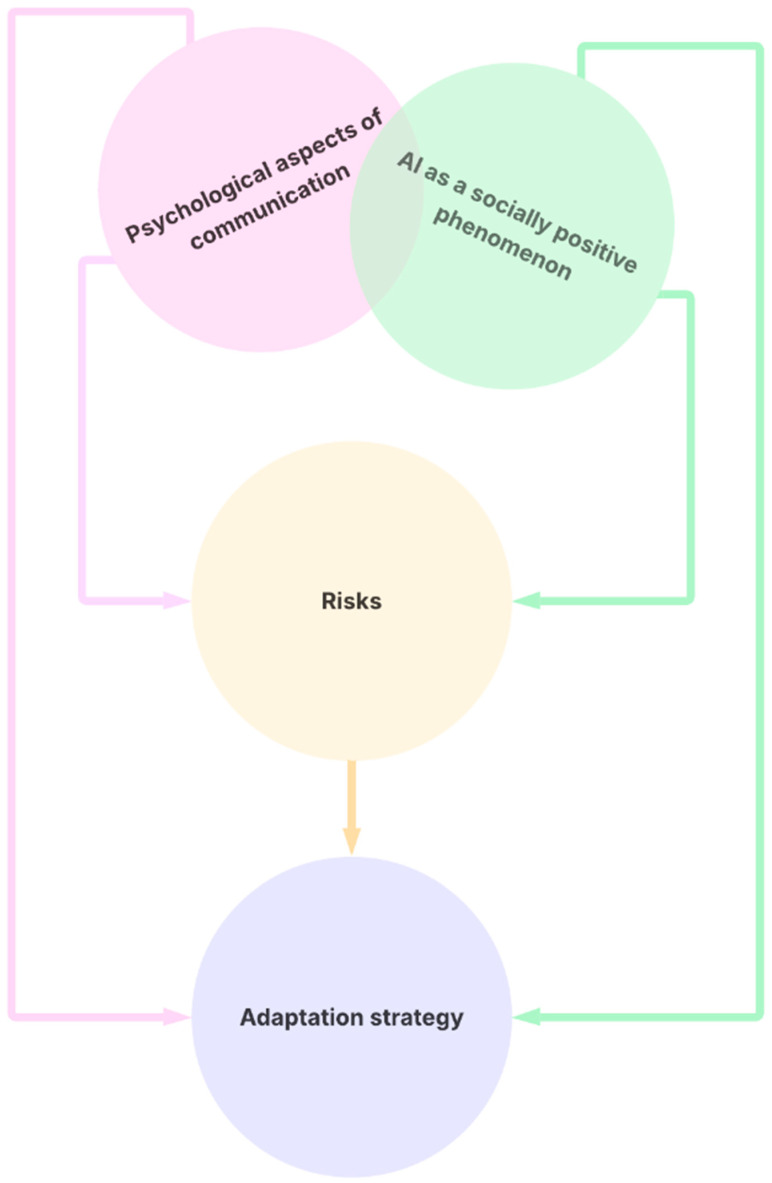
Abstracting individual topics into an interaction diagram of the digital wellbeing of human and AI interactions. The diagram shows the relationships between separate objects constituting the mechanisms of digital wellbeing formation.

In this ambivalent field, digital wellbeing can be understood as a phenomenon of dynamic equilibrium. However, these areas are not separate; they cannot be understood as isolated entities, but as a dynamic structure with interconnections, as shown in Figure 2. This dynamic description can provide a better idea of what digital wellbeing means in the context of artificial intelligence. Figure 2 provides a simplified representation of the model presented in Figure 1. It shows that internal (psychological-communicative) and external (socio-cultural) influences form a single field. This field gives rise to perceived risks and adaptation strategies resulting from critical reflection on risks and socio-cultural-psychological determinants. Once again, a pragmatic perspective is applied, emphasising the importance of a holistic view of human thought and behaviour within a given environment. This environment is shaped, perceived and interpreted by humans selectively.

## 4. Discussion

The research question of this study was as follows: “How do LIS students reflect on the relationship between their digital wellbeing and generative AI?”. A synthesis of the extant literature reveals an ambivalent consensus regarding the nature of these reflections. On the one hand, they perceive it as an extension of their capabilities, which can assist them in achieving their objectives, succeeding in competition, or—as Burr and Floridi (2020a) assert—leading a good life. Concurrently, however, they fundamentally identify threats that may have been part of the previous technological discourse or information revolution (Bridle, 2018; Webster, 2014), but which take on a new dimension or meaning in the context of generative AI (Floridi, 2025; Floridi & Chiriatti, 2020). It is important to note that statements which are made at different times may contain a completely different depth of concern or experience. In this specific context, they perceive digital wellbeing as a form of balancing between the social environment and the concerns they are aware of.

This necessitates the exploration of novel methods of communication with these systems, which are uncharted territory in this domain. However, it is important to note that this does not imply that these methods are infallible or without flaws. This ability to conceptualise communication in new ways, in the broad tradition of the philosophy of dialogue (Buber, 2017), represents a starting point for concrete adaptation mechanisms. The insight gained from established theory has allowed us to understand that digital wellbeing in this area is not a matter of fixed approaches or measures, but a truly dynamic process (Vanden Abeele, 2021) that is realised at the individual level, yet with a strong interactive link to technological development and social pressure.

The research conducted leads to the development of a basic conceptual framework for reflecting on digital wellbeing about generative artificial intelligence as a kind of interconnected four-dimensional model, which is captured in Figure 1. It combines sociological influences—namely, the perception of AI as a socially wanted and supported phenomenon that cannot be avoided, and the psychological aspects associated with interacting with a particular inhuman and untrustworthy actor. It offers the lure of expanding human capabilities and performance. These sociological-psychological aspects are then responded to by two other interrelated areas—identifying risks (challenges and opportunities already embedded in the social context) and adaptation strategies as coping with the phenomenon. Digital wellbeing can then be understood in the context of generative AI as achieving a balance between these four areas, which strongly influence each other.

Regarding AI as a specific phenomenon desired by society, students talk about the need to work effectively with AI and the ability to define appropriate tasks or skills related to creating prompts. This notion corresponds well to a confident positive attitude towards AI (Eager & Brunton, 2023; Fauzi et al., 2023), including the recognition of its limits (Theophilou et al., 2023) or working with it in specific areas of education (Heston & Khun, 2023). A separately discussed topic is the productivity of working with AI, where studies highlight both performance and productivity gains, but with the need to observe long-term impacts, for which there is not yet enough data (Al Naqbi et al., 2024; Damioli et al., 2021). It is the long-term perspective that is crucial in the context of educational practice.

The second area that our research identified regarding digital wellbeing was the psychological aspects of communicating with generative AI. Students emphasise the need to distinguish between AI and humans when talking to others and interacting with technology, however difficult this distinction is for them (Condrey, 2023; Schober, 2022). Although seemingly inconsequential, text-based communication, characterised by its formal features that echo human communication, constitutes a foundational element in the design of generative AI (Floridi, 2023a, 2023b; Loos et al., 2023). However, it is also central to the intricate reflection on the “intelligence” and authenticity of communication with generative AI systems. The situation is formally analogous to that with older forms of technology (Bridle, 2018; Heidegger, 1967b), yet fundamentally novel.

At the same time, it turns out that there is also no single narrative—artificial intelligence can act as a tool (Sanji et al., 2022), an assistant (Deepika et al., 2020), or a non-human (Floridi, 2025). At the same time, communication is specific because it does not have the character of a human dialogue, lacking the reflexivity of truthfulness (Elias & Alija, 2023) or accountability (Floridi, 2023b). Students are thus thrown into a form of collaboration or dialogue (Aboelmaged et al., 2024; Buber, 2017; Clarizia et al., 2018), which, on the one hand, exhibits human features of communication and, on the other hand, is radically different.

The third dimension of the model is perceived risks. These cover the commonly identified problems mentioned in the context of AI relatively well. Students talk about environmental factors (Zewe, 2025), error rates and hallucinations (Alkaissi & McFarlane, 2023; Lin et al., 2021), security issues (Pasupuleti et al., 2023), copyright protection (Lucchi, 2024; Pasupuleti et al., 2023), the need to verify information (Amaro et al., 2023), hate content (Louati et al., 2024), or addictive behaviour (Ferreri et al., 2018; Montag et al., 2025). What emerged as necessary for the research was not simply the analysis of possible risks, but the way they are perceived—students are aware that risks are there and that they have to navigate in this field of risks, so risks form a kind of environment (Johnson, 2017; Lakoff, 1990) in which a whole mental conceptualisation of relationships to technology emerges (Burr & Floridi, 2020a). It is not about external academically analysed risks, but a description of the environment in which students live and think, of the problems they experience in interacting with AI. It seems educationally necessary to make this reframing of the understanding of the risks of AI and how they affect students in the university environment.

The fourth area is adaptation strategies. In some respects, the three previous areas can be understood as a form of phenomena less dependent on the students themselves, which affect their wellbeing both through the creation of the external environment and through the process of internalisation (Figure 2), that is, as the role of the environment is understood in pragmatism (Johnson, 2007, 2017; Lakoff & Johnson, 2003), which imprints itself on forms of thought. To some extent, these forms of thinking in students fulfil Heidegger’s question after technology (Heidegger, 1967b): what can we do to not live in the drag of technology in such an environment, to preserve specific vital parameters of humanity? In terms of the impact on the educational environment, it will be necessary in the future to assess whether (or to what extent) the particular elements that students write about fulfil the role of a specific effort to romantically rescue humans from technology, and to what extent they are the source of the formation of a particular new robust conception of humanism forming a certain new starting point for active strategies for achieving digital wellbeing in the context of generative artificial intelligence. It attempts to achieve a dynamic balance in an environment (Floridi, 2013, 2014) with a new (essentially threatening) actor (Floridi, 2014). However, we must be careful not to make excessive generalisations—our data reflect how LIS students think, act, and reflect.

The key adaptive strategy students describe is the ontological distinction between AI and humans (Floridi, 2023b)—an apparent abandonment of the information agent narrative (Floridi, 2014) that was present in public debate before the massive spread of generative AI. Students emphasise the human aspects of communication, such as responsibility (Coeckelbergh, 2023; Santoni de Sio & Mecacci, 2021) or the philosophically and psychologically crucial dimension of “caring or caring for the other” (Liu, 2024). Students try to emphasise the importance of preserving autonomy and freedom of decision (Bridle, 2018; Heidegger, 1967a), which they feel is threatened by AI (Pöhler et al., 2025). They then specifically emphasise two forms of thinking—critical thinking (Spector & Ma, 2019) and creativity (Ali Elfa & Dawood, 2023; Grassini & Koivisto, 2025).

A key issue is the discussion around the relationship between digital wellbeing and ethics (Burr & Floridi, 2020b), which for students is not just seen as a question of limits, something they would like to do but cannot because it is not right, we can see this dimension in the discussion around plagiarism with AI (Dien, 2023), but primarily in terms of difference. The emphasis on transparency in the use of AI (Walmsley, 2021), which this study has uncovered, is the transparency of ontological difference, the process of explicating what is truly authentically human, what has value.

It is imperative to emphasise that generative AI is a constituent of the technological realm, with certain aspects distinctly associated with it. Additionally, some aspects can be interpreted as being generally pertinent to digital wellbeing, even before the advent of the AI revolution. In the present analysis, no distinction is made between these two aspects, which are available in analytical insight, because they are perceived as an integral part of the infosphere (Floridi, 2014). While it is analytically possible to disaggregate these elements, such an approach obscures the intricate interconnections and holistic integration that characterise the environment in which the students formulated their recommendations. Formally identical statements—such as replacing interpersonal relationships—can have a completely different meaning in the context of generative AI than without it.

### Research Limitations and Ethics

The limitations of the research can be seen primarily in the limited sample size—we worked with assignments from 61 students, representing a limited number of responses. However, this is sufficient for qualitative research, as we conducted it in this study. Our aim in this study is not to describe student wellbeing, but to offer insights into the formation of its theoretical conceptualisation.

This is related to the second limit, which is the nature of the sample. By working with data from LIS students at one university and in one year of study, there was undeniably the creation of a specific discourse grasp linked to the influence of the entire social group and educational programme on the nature of the responses. On the one hand, this is true; on the other hand, these are students who have received a specific educational training that has enabled them to formulate responses of a higher quality or expertise than the general population could work with, but this does not mean that they have not also experienced similar elements structurally. At the same time, it cannot be said that these students were completely isolated from the cultural space in which they were situated.

Although the individual elements (external entities) in Figure 1 are diverse and largely dependent on the students’ individual experiences, the model created based on four interacting parts could have more general significance for all university students. This statement is based on the saturation of the sample and the degree to which these categories are general. However, given the research design, this claim can only be formulated as a hypothesis requiring quantitative verification on a broader research sample.

In general, these are issues that are associated with qualitative research. Our research design is not quantitative; as much as we work with 474 research units, the research is consistently qualitative, which we consider essential in studying this phenomenon. It is also undeniably strongly influenced (through the first two parameters) by the cultural climate, with the educational focus of the study programme reflecting a particular environment in the Czech space that is generally less critical of generative AI than is common in, for example, Western Europe. This fact may lead to a certain skewing of responses (systematic error), which we do not have control over—both towards optimism (taking over the narrative) and pessimism (leaning towards Western rather than Eastern discourse in the European space). Again, this limit does not constitute a significant obstacle by not examining the contribution of particular phenomena in society, but by forming a specific theoretical model. Still, it is necessary to reflect it in data interpretation.

The third limitation is related to the fact that this was a homework exercise—some of the tasks could have been created using ChatGPT, Gemini or another generative tool. It should be said here that students sometimes acknowledge using these tools in the sources for their rules and list them among the sources. Nevertheless, it can be said that if they select the rules they do, it reflects their own needs, preferences, values and interests. Because this was a homework assignment, it is also possible that not all students state their attitudes and opinions accurately, or that they perceive the topic as relevant and vital. We try to minimise this parameter by using the interpretive-analytical method.

The fourth limitation relates to the research itself. There may be aspects or phenomena important for digital wellbeing concerning generative AI, but which cannot be translated into rules. We partially overcome this limitation by conducting a thematic analysis within a pragmatic philosophical framework, allowing for relatively free transitions between thinking and acting, rationality and emotionality, and the explicit and implicit analysis of experience. However, the high degree of abstraction in the models (Figure 1 and Figure 2) obscures some possible sub-aspects we cannot capture. These could, however, be reflected in the partial entities in the outer circle around the four dimensions in the model, and of course, in the results themselves. Here, the research design is limited, as it works with a relatively large sample of individuals and statements, which restricts the scope for individual immersion.

The fifth limitation of the research design is that the coding was performed by a single researcher (repeatedly and at intervals). However, given the strictly qualitative nature of the study, this is not a limitation that would affect the relevance or reliability of the research.

Regarding research ethics, the tasks were collected to form a single set with indistinguishable responses from individual learners before coding began. The files were downloaded from the information system, mixed, transcribed (from Czech, Slovak and English) and then inserted into the analysed document. Due to the similar nature of the individual responses, the researcher cannot identify individual learners. It is therefore a procedure between fully and semi-anonymous data processing, which fully protects the privacy of the respondents. As reported in the Results section, the responses are after English proofreading, so they have undergone some sub-stylisation, again anonymising individual responses. No personal information or data in the responses would allow the identification of a student or a group of students.

Given the anonymous nature of the data processed and the absence of sensitive or personal data, this area does not fall within the remit of the ethics committee (Masarykova univerzita, n.d.). The data processing method also considers individual elements that do not allow for identifying specific students or their identification in any way. There was no direct interaction with the participant. The entire data processing process was set up to ensure the complete anonymity of all students whose text responses in the form of decalogues we analysed. The study was conducted transparently, with data privacy and anonymity as per the Helsinki Declaration.

## 5. Conclusions

This study focuses on a highly novel phenomenon that has lacked theoretical conceptualisation. This study uses thematic analysis and grounded theory methodologies to present an original perspective on how LIS students conceptualise digital wellbeing in the context of generative artificial intelligence. The present study explores a subject that is both dynamic and changeable, with these parameters exerting a significant influence on the conclusions. Digital wellbeing in the context of generative artificial intelligence is highly sensitive to environmental, cultural, and technological changes (Vanden Abeele, 2021), as well as to social pressure and the respondents’ ability to critically self-reflect on their relationship to this technology. The study uses a pragmatic philosophy approach to formulate a model that can serve as a basis for further psychological research or educational interventions. The research builds on Vanden Abeele’s (2021) conceptual foundations of dynamically understood digital wellbeing. Thanks to the research method and the combination of digital wellbeing and generative artificial intelligence, it also significantly develops and supplements it.

The research highlighted the interconnectedness of the sub-phenomena and the fact that the different components of digital wellbeing cannot be separated from each other, while at the same time identifying four key areas, its constituencies—the socio-cultural-educational climate creating the need to work with AI; the need to find new communication schemes with technical systems that are externally communication-like to humans but having a fundamentally different ontology; the need to live in the space of risks associated with AI and the formation of various adaptation strategies. Research has shown that it is essential to work with both risks and adaptive strategy in a new way in the educational space—it is necessary to integrate them into a reflective environment in which students achieve equilibrium. Risks are not external descriptions or remote threats, but phenomena that students have to cope with in their environment.

LIS students comprehensively understand the principles that govern digital wellbeing in the context of generative AI. This understanding is characterised by a nuanced appreciation of the balance between these technologies’ potential risks and threats and the social pressures that encourage their utilisation. Digital wellbeing is therefore regarded as a balance, rather than as relinquishing the use of technology or a series of prohibitions.

It is impossible to assess individual adaptation strategies independently and evaluate their adequacy or functionality “on their own” (atomically). The study demonstrates that this atomistic conceptual construct is inadequate in expressing the complexity of the contemporary world. This corresponds well with the research data and the philosophical foundations of pragmatic philosophy (Damasio, 2018; Johnson, 2017; Lakoff, 1990).

It is essential to look for educational approaches that allow students to learn to interact more adequately with AI (the question is whether it will be necessary to change the structure of terminology such as intelligence or communication about technology), while at the same time opening up a fundamentally deeper discussion about what is “authentically human” and systematically promoting these elements in the context of education. The students’ answers also clearly showed a demand for the artificiality or appropriateness of generative AI in education and the formation of specific rules and limits in this field. Grand claims regarding authenticity, transparency, autonomy, and humanity are integral to the discourse within the students’ statements, a phenomenon unmistakably associated with their academic pursuits at the faculty of arts. It may be hypothesised that students of more technical fields would adopt a more pragmatic approach; however, this does not necessarily imply any causal relationship between this and the fundamental patterns described in Figure 1 and Figure 2.

This practical educational psychological research area should be the focus of further research activities. The whole topic is still new, poorly described, and above all, lacks the perspective of a longer-term school studying the effects of AI on digital wellbeing from the standpoint not of weeks or months, but of years. In this context, it is therefore essential, as this research has done, to bring together educational, psychological and philosophical approaches to the analysis of this phenomenon. This model, delineated in this study, can be a foundational framework for subsequent investigations.

## Figures and Tables

**Table 1:** The table shows a description of the codes’ basic structure and frequency.

Categories	Description	Frequency	Subcategories
AI literacy	Topics related to understanding the principles of how AI works, how to use it effectively, and learning how to work with AI.	60	Effectiveness and prompting (20), AI principles (17), education (17), and miscellaneous (8).
Copyright	Topics related to copyright protection and copyright abuse by AI tools.	17	
Security—general	General security features that could not be classified as data and information protection. This includes, for example, the choice of passwords for services.	9	
Uncategorized	Statements that could not be classified in any of the categories	10	
Environmental factors	Statements related to limitations on using AI concerning ecological and environmental factors.	44	
Ethics	Rules relating to various aspects of ethical work with generative AI—on transparency and explication of use, plagiarism, creation of false, hateful or mendacious content, and other elements.	75	Hateful and false content (24), plagiarism (8), transparency (24), and miscellaneous (23)
Thinking	Statements related to transforming thinking, especially creativity, critical thinking, the limits of AI, themes of accountability, and understanding AI as a facilitator	91	AI as an enabler (13), creativity (6), critical thinking (30), limits of AI (19), responsibility (17), miscellaneous (6).
Data and information protection	Data and information protection rules, especially at the input level (not giving out personal data of oneself or others, passwords, etc.).	49	
Verification of information	Topics related to the need to verify information obtained because of its low or questionable reliability.	47	
Wellbeing	Topics related to wellbeing itself, mainly referring to the need to set time and other limits, that AI is not human, and AI must have other kinds of interactions, but also other specific measures	72	Time and boundaries (39), inhuman actors (17), other specific measures—outside of time (8), and miscellaneous (12)

**Table 2:** The table shows common statements across multiple categories. Items marked with an X indicate intersections of identical categories; items without numbers correspond to zero intersections.

	AI Literacy	Copyright	Data and Information Protection	Environmental Factors		Ethics	Security—General	Thinking	Uncategorized	Verification of Information		Wellbeing
**AI literacy**	X							2	6	3	2	1
**Copyright**			X									
**Data and information protection**				X				1			1	
**Environmental factors**					X							
**Ethics**						X			4			
**Security—general**	2			1				X	1		1	
**Thinking**	6					4		1	X	1	2	8
**Uncategorized**	3								1	X		
**Verification of information**	2			1				1	2		X	1
**Wellbeing**	1								8		1	X

## Data Availability

Data sharing not applicable. No new data were created or analysed in this study. Data sharing does not apply to this article.
